# Effects of *FTO* RS9939906 and *MC4R* RS17782313 on obesity, type 2 diabetes mellitus and blood pressure in patients with hypertension

**DOI:** 10.1186/1475-2840-12-103

**Published:** 2013-07-12

**Authors:** Aline Marcadenti, Flavio D Fuchs, Ursula Matte, Fernanda Sperb, Leila B Moreira, Sandra C Fuchs

**Affiliations:** 1 Postgraduate Studies Program in Cardiology, School of Medicine, Universidade Federal do Rio Grande do Sul, R. Ramiro Barcelos 2600, 2º. andar, Porto Alegre, RS, Brazil; 2 Division of Cardiology, Hospital de Clínicas de Porto Alegre, and the National Institute for Science and Technology for Health Technology Assessment (IATS/CNPq), R. Ramiro Barcelos 2600, 2º. andar, Porto Alegre, RS, Brazil; 3 Center for Gene Therapy, Hospital de Clínicas de Porto Alegre, R. Ramiro Barcelos 2600, 1º. andar, Porto Alegre, RS, Brazil

**Keywords:** Diabetes mellitus, Hypertension, *FTO* rs9939609, *MC4R* rs17782313, Waist circumference, Neck circumference, Lipid Accumulation Product index, Body mass index

## Abstract

**Background:**

Genetic variants of the *FTO* gene rs9939609 A/T and the *MC4R* gene rs17782313 C/T have been associated with obesity. Individuals with mutations in *MC4R* gene have lower blood pressure (BP), independently of obesity. This study aimed to investigate the association of *FTO* rs9939609 and *MC4R* rs17782313 with anthropometric indexes, BP, and type 2 diabetes mellitus among hypertensive patients.

**Methods:**

We genotyped 217 individuals (86 men and 131 women) with hypertension (systolic or diastolic BP ≥ 140/90 mmHg or using antihypertensive drugs). Diabetes mellitus was diagnosed according to the American Diabetes Association criteria. Waist and neck circumferences (cm), Body Adiposity Index (BAI,%), Lipid Accumulation Product Index (LAP, cm.mmol.l) and body mass index (BMI, kg/m^2^) were analyzed using analysis of covariance or modified Poisson’s regression.

**Results:**

Rare allele frequencies were 0.40 for A for *FTO* rs9939609 and 0.18 for C for *MC4R* rs17782313. A positive association of *FTO* rs9939609 and *MC4R* rs17782313 with BMI was observed in the overall sample. Among men and women, neck circumference was associated with the *FTO* genotype and, for women, *MC4R* genotype. In contrast, in men we found a negative association of *MC4R* rs17782313 with diastolic BP (TT 90.1 ±12.2, TC/CC 83.2 ±12.1; P = 0.03) and borderline association for systolic BP after controlling for age and BMI.

**Conclusions:**

Common genetic variants of *FTO* rs9939609 have positive associations with BMI and neck circumference and *MC4R* rs17782313 in women, but a negative association with diastolic and mean blood pressure in men with hypertension.

## Introduction

Obesity is a well-known risk factor for hypertension and type-2 diabetes mellitus [1]. Genetic factors play an important role in the development of obesity [2], but the underlying genetic pathways are poorly understood.

*FTO* (*fat mass and obesity*-*associated*) gene was first identified in mouse [3] and it has been associated with energy balance [4,5]. *MC4R* (*melanocortin 4 receptor*) gene was the first loci in which mutations were associated with morbid obesity in human beings [6,7]. The most common genetic variant of the *FTO* gene (rs9939609 A/T) [8] and a variant near the *MC4R* gene (rs17782313 C/T) [9] were detected recently and both single nucleotide polymorphisms (*SNP*s) have been associated mainly with overall [8,9] and, in some cases, abdominal obesity [10-12]. Cardiovascular risk factors as type-2 diabetes mellitus [13,14], insulin resistance [15], and hypertension [16] were associated with the risk allele A for *FTO* rs9939609 and the risk allele C for *MC4R* rs17782313, regardless body mass index [13,14].

*FTO* gene is strongly expressed in hypothalamus [17,18], a brain structure involved in the control of energy homeostasis [5] and regulation of blood pressure [19]. The association between the risk allele A for *FTO* rs9939609 polymorphism and elevated blood pressure was demonstrated in Caucasian populations [16]. *MC4R* gene is also expressed in the hypothalamus [18,20], but when inactivated is associated with lower blood pressure, independently of obesity [21]. However, this association has been shown only in individuals with mutations [21]. In the overall population, individuals carrying the risk allele C for near *MC4R* rs17782313 did not have different blood pressure levels comparing to non-carriers [22]. For type-2 diabetes mellitus risk, studies that evaluated both polymorphisms in the overall population are inconclusive [8,23,24].

Among individuals with hypertension, who are often obese and have type-2 diabetes [25], the contribution of both polymorphisms on obesity, blood pressure control and type-2 diabetes mellitus had not been investigated. The aim of this study was to investigate the association between *FTO* rs9939609, *MC4R* rs17782313 with anthropometric indicators, blood pressure, and type-2 diabetes in patients with hypertension.

## Methods

This is a cross-sectional study, which enrolled men and women, aged 18 to 80 years, who had been diagnosed with hypertension, living in the urban area of Porto Alegre, southern Brazil. In total, a sub-sample of 217 patients was genotyped (38.8%) and examined in an outpatient clinic of a reference hospital. The study was approved by the Ethical Committee of our institution, and all patients accepted to participate and signed a consent form.

### Genotyping

DNA was extracted from peripheral blood using NucleoSpin^®^ Blood (Macherey-Nagel GmbH & Co. KG, Germany). StepOne^TM^ Real-Time PCR Systems (Applied Biosystems Inc., Foster City, CA) was used to genotype the *FTO* locus rs9939609 and the *MC4R* locus rs17782313 using TaqMan-based assays (Applied Biosystems Inc., Foster City, CA).

### Outcome variables

Trained research assistants carried out six standardized blood pressure measurements [26], obtained in three visits, using an aneroid sphygmomanometer with an adequate sized cuff to the arm circumference and the average was used to calculate mean blood pressure [(Systolic BP + 2*Diastolic BP)/3]. Hypertension was diagnosed by systolic blood pressure ≥140 or diastolic blood pressure ≥90 mmHg, or use of antihypertensive lowering agents [27]. Type-2 diabetes mellitus was diagnosed by fasting blood glucose ≥ 126 mg /dl, glycated hemoglobin ≥ 6.5%, or use of anti-diabetic agents [28].

### Other covariates

Demographic data and information regarding education (years at school), smoking (ex-smoker or current smoker), abusive alcohol consumption (≥30 g ethanol/day in men and ≥15 g/day in women), and physical activity (short version of the *International Physical Activity Questionnaire*, IPAQ [29], categorized as low/moderate or high intensity) were collected using standardized questionnaires by trained interviewers.

Weight (kg) was measured with patients in light clothes, barefoot, in a 100 g scale (Filizola^®^, model 31, IN Filizola – SA, Sao Paulo, Brazil) and height was obtained with patient in Frankfurt plane, using a Tonelli^®^ stadiometer with 0.1 cm scale, model E120 A (IN Tonelli – SA, Santa Catarina, Brazil). Body mass index (BMI) was calculated by the weight (kg)/height (m)^2^.

Waist circumference was obtained with a plastic, flexible, inelastic measuring tape positioned in the middle point, between the last costal rib and the iliac crest, in a perpendicular plane, with patient standing in both feet, approximately 20 cm apart, and with both arms hanging freely at the side, with palms facing thighs. Neck circumference was measured with the head straight and eyes staring forward, horizontally, one inch below the laryngeal prominence, and hip circumference at the level of the higher diameter over the buttocks. The anthropometry was carried out in duplicate, with a one week break, by interviewers trained independently and periodically reassessed. Body Adiposity Index (BAI) was calculated using the formula: hip (cm)/[height (m)^1.5^]-18 [30].

Serum triglycerides were measured by calorimetric enzymatic method using standardized techniques. Lipid Accumulation Product Index (LAP) was calculated from waist circumference, in centimeters, plus serum triglycerides, in mmol, for men [(WC-65)*TG] and women [(WC-58)*TG] [31].

### Analyses

Statistical analyses were performed using SPSS (*Statistical Package for the Social Science*, SPSS^®^, version 17.0, IL, USA). Means ± SD and percentages were compared using analysis of variance or Pearson’s chi-square test. We tested the potential relationship of genotype and blood pressure, using analysis of covariance models, with the adjustment for age and BMI. Modified Poisson’s regression (expressed as risk ratio and 95% CI) was used to analyze the association between genotype and type-2 diabetes, after adjustment for age and BMI. A P value between 0.05 and 0.1 was considered as a trend toward association and less than 0.05 as statistically significant.

## Results

Characteristics of individuals included in the study for each polymorphism are shown in Table 1. Rare allele frequencies were 0.40 for A for *FTO* rs9939609 and 0.18 for C for *MC4R* rs17782313. Genotype frequencies did not differ from those predicted by the Hardy-Weinberg equilibrium (P = 0.8 for *FTO* and P = 0.8 for *MC4R*).

**Table 1:** **Characteristic of participants according to polymorphism** [**mean** ± **SD**, **n** (%)]

	**rs9939609**			**rs17782313**		
	**TT**	**TA/AA**	**P value**	**TT**	**TC/CC**	**P value**
	**(n = 75)**	**(n = 142)**		**(n = 146)**	**(n = 71)**	
Age (years)	59.6 ±11.4	60.2 ±11.1	0.7	60.3 ±11.3	59.2 ±10.8	0.5
Sex			0.9			0.02
Men	30 (34.9)	56 (65.1)		66 (76.7)	20 (23.3)	
Women	45 (34.4)	86 (65.4)		80 (61.1)	51 (38.9)	
Vigorous physical activity			0.7			0.2
Yes	11 (31.4)	24 (68.6)		27 (77.1)	8 (22.9)	
No	64 (35.4)	117 (64.6)		118 (65.2)	63 (34.8)	
Body mass index (kg/m^2^)	28.9 ±4.6	30.7 ±5.2	0.02	29.6 ±4.8	31.1 ±5.4	0.04
Waist circumference (cm)	99.5 ±11.5	102.1 ±11.6	0.1	100.3 ±11.2	103.1 ±12.1	0.1
Neck circumference (cm)	37.7 ±3.4	38.9 ±3.8	0.03	38.5 ±3.8	38.5 ±3.3	0.9
Body Adiposity Index (%)	33.6 ±5.8	34.9 ±6.9	0.2	33.9 ±6.5	35.7 ±6.6	0.07
LAP^*^ (cm.mmol.l)	4.1 ±0.8	4.2 ±0.6	0.4	4.1 ±0.6	4.2 ±0.7	0.2
Type-2 Diabetes			0.7			0.07
Yes	32 (33.0)	65 (67.0)		59 (60.8)	38 (39.2)	
No	43 (35.8)	77 (64.2)		87 (72.5)	33 (27.5)	
SBP^§^ (mmHg)	155.2 ±22.7	150.8 ±19.7	0.1	154.3 ±21.8	147.9 ±18.1	0.04
DBP^¶^ (mmHg)	88.9 ±13.2	87.1 ±13.1	0.3	88.5 ±13.4	86.1 ±12.4	0.2
Mean blood pressure (mmHg)	111.0 ±14.1	108.3 ±13.1	0.2	110.5 ±13.9	106.7 ±12.3	0.06

*LAP Lipid Accumulation Product, log-transformed; § systolic blood pressure; ¶ diastolic blood pressure.

In total, 86 men and 131 women were assessed, aged 59.8 ± 11.2 years, systolic blood pressure of 152.1 ± 20.6 mmHg, and diastolic blood pressure of 87.7 ± 13.0 mmHg. The prevalence of type-2 diabetes mellitus was 44.7%; 4.2% of the sample reported abusive alcohol consumption and 43.8% were current or ex smokers.

### Obesity

*FTO* rs9939609 individuals with AT and AA genotypes had higher BMI than those with TT, as well as those who had *MC4R* rs17782313 with CT and CC genotypes. In addition, neck circumference was higher among AT/AA carriers than TT carriers according to *FTO* risk allele. For other anthropometric indices, the P values remained as a trend toward association, as seen for waist circumference (*FTO* and *MC4R*) and Body Adiposity Index (BAI).

Analyses conducted according to gender (Table 2) showed that men and women who were carriers of AT/AA genotype had higher neck circumference than those homozygotes TT for *FTO* rs9939609, while a trend toward association was verified for body mass index. Women carrying risk alleles AA/AT also had higher Body Adiposity Index versus TT for *FTO* genotype.

**Table 2:** **Characteristic of men and women according to polymorphism risk allele A for *FTO *rs9939609** [**mean** ± **SD**, **n** (%)]

	**Men**			**Women**		
	**TT**	**TA/AA**	**P value**	**TT**	**TA/AA**	**P value**
	**(n = 30)**	**(n = 56)**		**(n = 45)**	**(n = 86)**	
Age (years)	63.9 ±8.8	58.9 ±11.1	0.03	56.7 ±12.2	60.9 ±11.1	0.04
Body mass index (kg/m^2^)	28.3 ±4.4	29.7 ±4.1	0.1	29.5 ±4.7	31.3 ±5.8	0.07
Waist circumference (cm)	101.3 ±12.4	104.1 ±10.7	0.3	98.2 ±10.8	100.9 ±11.9	0.2
Neck circumference (cm)	40.5 ±3.0	41.7 ±2.6	0.04	35.9 ±2.8	36.9 ±3.1	0.04
Body Adiposity Index (%)	29.4 ±3.2	29.4 ±3.5	0.9	36.4 ±5.3	38.6 ±6.1	0.04
LAP^*^ (cm.mmol.l)	4.1 ±0.8	4.3 ±0.6	0.2	4.1 ±0.7	4.1 ±0.6	0.9
SBP^§^ (mmHg)	155.2 ±22.5	150.1 ±19.9	0.3	155.1 ±23.1	151.2 ±19.6	0.3
DBP^¶^ (mmHg)	88.5 ±14.7	88.5 ±11.6	0.9	89.4 ±12.2	86.3 ±13.9	0.2
Type-2 Diabetes			0.9			0.5
Yes	14 (35.9)	25 (64.1)		18 (31.0)	40 (69.0)	
No	16 (34.0)	31 (66.0)		27 (37.0)	46 (63.0)	

*LAP Lipid Accumulation Product, log-transformed; § systolic blood pressure; ¶ diastolic blood pressure.

According to *MC4R* rs17782313 genotype, there were no significant differences among anthropometric indexes for men with CT/CC genotype, in comparison to homozygotes TT (Table 3). Waist circumference and Lipid Accumulation Product Index had a trend to be higher in women with CT and CC genotypes and neck circumference was significantly higher among women carrying risk allele C.

**Table 3:** **Characteristic of men and women according to polymorphism risk allele C for *MC4R *rs17782313** [**mean** ± **SD**, **n** (%)]

	**Men**			**Women**		
	**TT**	**TC/CC**	**P value**	**TT**	**TC/CC**	**P value**
	**(n = 66)**	**(n = 20)**		**(n = 80)**	**(n = 51)**	
Age (years)	61.1 ±10.3	59.2 ±11.3	0.5	59.7 ±12.1	59.2 ±10.8	0.8
Body mass index (kg/m^2^)	28.9 ±4.4	30.1 ±4.1	0.3	30.1 ±5.4	31.5 ±5.7	0.2
Waist circumference (cm)	102.5 ±11.1	105.1 ±11.9	0.4	98.5 ±11.1	102.3 ±12.2	0.07
Neck circumference (cm)	41.3 ±2.9	41.2 ±2.7	0.9	36.1 ±2.8	37.4 ±2.9	0.01
Body Adiposity Index (%)	29.4 ±3.4	29.4 ±3.5	0.9	37.6 ±6.0	38.1 ±5.9	0.7
LAP^*^ (cm.mmol.l)	4.2 ±0.7	4.3 ±0.8	0.9	4.0 ±0.6	4.2 ±0.7	0.07
SBP^§^ (mmHg)	154.3 ±21.2	143.8 ±18.1	0.04	154.3 ±22.4	149.7 ±17.9	0.2
DBP^¶^ (mmHg)	89.8 ±12.8	84.1 ±11.7	0.08	87.5 ±13.9	86.9 ±12.8	0.8
Type-2 Diabetes			0.9			0.02
Yes	30 (76.9)	9 (23.1)		29 (50.0)	29 (50.0)	
No	36 (76.6)	11 (23.4)		51 (69.9)	22 (30.1)	

* *LAP* Lipid Accumulation Product, log-transformed; § systolic blood pressure; ¶ diastolic blood pressure.

### Type-2 diabetes mellitus

There was no difference in prevalence of type-2 diabetes according to *FTO* rs9939609 genotype. The trend toward association detected for *MC4R* rs17782313 (Table 1), further analyzed by gender showed that women who had *MC4R* rs17782313 CT/CC genotype had higher prevalence of diabetes than those with TT allele (Table 3). The multivariate analysis taking into account age and BMI showed that the allele C for *MC4R* was associated with higher risk of type 2 diabetes mellitus in women (RR = 1.5 95%CI: 1.1 – 2.2, P = 0.03), but not among men (RR = 0.9 95%CI: 0.6 – 1.7, P = 0.9). *FTO* was not associated to diabetes among men (RR = 1.1 95%CI: 0.7 – 1.7, P = 0.9) and women (RR = 1.0 95%CI: 0.7 – 1.6, P = 0.9).

### Blood Pressure

Carriers of risk alleles (A for *FTO* rs9939609 and C for *MC4R* rs17782313) had average diastolic blood pressure similar to non-carriers, but systolic blood pressure was significantly lower among *MC4R* rs17782313 individuals with CT/CC genotype and for *FTO* variant there was a trend toward association.

Table 4 shows systolic, diastolic, and mean blood pressure adjusted for age and BMI, among men and women according to the genotype. *FTO* was not associated with blood pressure for both genders, but among men with risk allele C for *MC4R* rs17782313, diastolic blood pressure was significantly lower (P = 0.03), and there was a trend for systolic blood pressure (P = 0.05). Therefore, mean blood pressure was markedly lower among those with allele C for *MC4R*, independently of age and BMI.

**Table 4:** **Systolic and diastolic blood pressure adjusted**-means* **among men and women according to genotype** [**mean** ± **SD**, (**95% CI**)]

	**Men**			**Women**		
**rs9939609**	TT	TA/AA	P value	TT	TA/AA	P value
	(n = 30)	(n = 56)		(n = 45)	(n = 86)	
Systolic BP (mmHg)	153.7 ±20.7	150.8 ±20.9	0.6	155.9 ±21.5	150.8 ±20.4	0.2
	(146.1 – 161.3)	(145.3 – 156.4)		(149.6 – 162.2)	(146.4 – 155.3)	
Diastolic BP (mmHg)	89.9 ±12.5	87.7 ±12.7	0.5	87.5 ±12.7	87.2 ±12.1	0.9
	(85.4 – 94.4)	(84.5 – 91.0)		(83.7 – 91.2)	(84.6 – 89.9)	
Mean BP (mmHg)	111.1 ±13.7	108.8 ±13.5	0.5	110.6 ±13.7	108,2 ±13.7	0.5
	(106.2-116.0)	(105.2-112.3)		(106.5-114.7)	(105,3-111,2)	
**rs17782313**	TT	TC/CC	P value	TT	TC/CC	P value
	(n = 66)	(n = 20)		(n = 80)	(n = 51)	
Systolic BP (mmHg)	154.2 ±20.3	144.2 ±20.1	0.05	154.1 ±20.6	150.2 ±20.7	0.3
	(149.2 – 159.1)	(135.3 – 153.2)		(149.5 – 158.6)	(144.3 – 155.9)	
Diastolic BP (mm Hg)	90.1 ±12.2	83.2 ±12.1	0.03	87.5 ±12.5	86.9 ±12.9	0.8
	(87.2 – 92.9)	(77.9 – 88.5)		(84.8 – 90.2)	(83.5 – 90.5)	
Mean BP (mmHg)	111.3 ±13.5 (108.1-114.6)	103.9 ±13.4 (98.0-109.8)	0.02	109.8 ±13.4 (106.8-112.7)	107.9 ±13.5 (104.1-111.7)	0.5

* Means adjusted for age and BMI.

## Discussion

As far as we know, this is the first study showing the association of a common genetic variant near *MC4R* gene with blood pressure in patients with hypertension. The negative association of *MC4R* common variant with blood pressure in men is a novelty, as it had been described only in morbidly obese individuals with rare mutations in the *MC4R* gene and in animal models. The association was further evaluated in analyses by gender, showing that men carrying the C allele for *MC4R* had lower diastolic and mean blood pressure compared with TT homozygotes.

Genome-wide association studies have been identified a number of genetic variants associated with obesity and their role in obesity-related vascular diseases [32]. Frayling et al. [8] showed for the first time the contribution of the *SNP* rs9939609 in *FTO* on excess of weight, and this result has been replicated in several populations [4,5,10,11]. As expected, common variant of *FTO* was associated with BMI in our overall population, but the analysis by gender confirmed only a trend toward association. Body Adiposity Index, a simple index which detects the percentage of total fat was significantly higher among women carrying *FTO* risk allele, and the effect of the gene on women body weight could be essentially over body fat. Among men, the association between *FTO* and BMI has not been confirmed [33] or it was weakened [34]. Another simple measure correlated with total and abdominal obesity is neck circumference [35], which was higher in men and women with risk allele A. This association has also been confirmed in European adolescents [36].

For the overall population, common variant near *MC4R* rs17782313 was associated with BMI and there was a trend to have subjects with higher BAI among CT/CC carriers. Analyses conducted by gender showed that in addition to the association of neck circumference with *MC4R* genotype, the trend toward higher waist circumference and Lipid Accumulation Product suggest an effect of the risk allele C on visceral obesity in women with hypertension. Mutation carriers on *MC4R* had increased lean mass [7] and the variant near *MC4R* could reflect this effect among in men, who had proportionally more free-fat mass comparing to women. Furthermore, men and women had differences regarding metabolism of free fatty acids, which increased accumulation on visceral adipose tissue in women when compared with men [37]. Among Chinese, no association was found between *MC4R* rs17782313 and abdominal obesity detected by waist circumference [38] and, in a European cohort, *MC4R* rs17782313 plus *FTO* rs1421085 were associated with abdominal obesity and metabolic syndrome. However, when analyzed separated, neither *MC4R* nor *FTO* were associated with any individual metabolic syndrome feature, including waist circumference [39].

In this study, type-2 diabetes mellitus was associated with women carriers of risk allele C for *MC4R* rs17782313, which remained significant after control for age and BMI. Studies that assessed the contribution of polymorphisms in *FTO* and *MC4R* genes on type-2 diabetes mellitus are controversial, probably due to ethnic differences regarding body composition and lifestyle among populations (significant differences about the frequencies of several common obesity susceptibility variants in or near *FTO* and *MC4R* were found in class III obese Hispanic patients, i.e.) [40]. Among hypertensive women, the effect of risk allele C for *MC4R* rs17782313 on type-2 diabetes mellitus could be mediated by increased visceral adipose tissue. *MC4R* showed higher expression in mesenteric fat among obese and diabetic rats when compared with lean rats [18]. Besides, studies support that the association of *FTO* rs9939609 and the *MC4R* rs17782313 polymorphisms with type-2 diabetes and obesity could be modulated and depended on diet [23]. It is also suggested that both type-2 diabetes and obesity had a common genetic etiology from the same 16.3 encoding region were *FTO* is located, and probably this is not the only susceptibility gene on this region which share the risk for both obesity and type-2 diabetes [41]. Genetic predisposition to obesity and *FTO* common variants contributed to increased insulin resistance among Europeans, but weakly increased type-2 diabetes risk, which is mediated by BMI itself [42].

As seen in morbid obese - with no hypertension and with rare mutations in *MC4R* gene, this sample of hypertensive individuals carrier of risk allele C for common variant near *MC4R* had lower levels of blood pressure in comparison to homozygote non-carriers. After adjustment for age and BMI, the association remained significant in men. It has been widely demonstrated in animal models the contribution of melanocortin system on cardiovascular function [43], and, in human beings, it has been shown how centrally expressed melanocortin 4 receptor (*MC4R*) not only conveys hypothalamic signals suppressing food intake, but it is also involved in blood pressure control [21]. Common variants near *MC4R* could reflect the same effect, but is unknown if anti-hypertensive drugs or changes in lifestyle could modify the expression of these genes or the effects of both polymorphisms on blood pressure, as it has been shown [44]. Among different model species, it has been demonstrated signaling cascades and transcription factors for several obesity genes that could be useful for development of new drugs and for translational research [45].

This study has some limitations and strengths that should be taken into account when interpreting the results. The analysis of genotype by gender is exploratory and it is underpowered to detect small differences. Therefore, some associations that achieved statistical significance in the overall analysis remained only as a trend toward association (BMI and *FTO* genotype, for instance). In the other side, some associations remained significant, as neck circumference and *FTO* genotype, while others came up only for women (*FTO* and BAI). The associations of gene variants with anthropometric indexes should be confirmed in further studies, with statistical power to carry out analysis by gender.

In conclusion, this study shows a negative effect of *MC4R* rs17782313 on mean arterial pressure and diastolic blood pressure among men. Associations of some anthropometric indexes with polymorphisms are exploratory for both, men and women. The pathways for these associations are not completely understood, but genetic variants near the *MC4R* gene may reflect the complexity of such mechanisms.

## Abbreviations

BAI: Body Adiposity Index; BMI: Body mass index; BP: Blood pressure; CI: Confidence interval; DNA: Deoxyribonucleic acid; FTO: Fat mass and obesity-associated; IPAQ: International Physical Activity Questionnaire; LAP: Lipid Accumulation Product Index; MC4R: Melanocortin 4 receptor; RR: Risk ratio; SNP: Single nucleotide polymorphism; TG: Triglycerides; WC: Waist circumference.

## Competing interest

No potential competing interest relevant to this article were reported.

## Authors’ contributions

AM, LBM, FDF, SCF have made substantial contributions to conception and design; AM, USM, FS, SCF analyzed and interpreted the data; AM, LBM, FS have been involved in drafting the manuscript, and USM, FDF, SCF in the reviewing it critically for important intellectual content. The authors had full access to all of the data in the study and AM and SCF take responsibility for the contents of the article. All authors have given final approval of the version to be published.

## References

[B1] Tuck ML Corry DB Prevalence of obesity, hypertension, diabetes, and metabolic syndrome and its cardiovascular complications Curr Hypertens Rev 2010 6 73 82 10.2174/157340210791171010

[B2] Rankinen T Zuberi A Chagnon YC Weisnagel SJ Argyropoulos G Walts B Pérusse L Bouchard C The human obesity gene map: the 2005 update Obesity (Silver Spring) 2006 14 529 644 10.1038/oby.2006.71 16741264

[B3] Van der Hoeven F Schimmang T Volkmann A Mattei MG Kyewski B Ruther U Programmed cell death is affected in the novel mouse mutant Fused toes (Ft) Development 1994 120 2601 2607 7956835 10.1242/dev.120.9.2601

[B4] Cecil JE Tavendale R Watt P Hetherington MM Palmer CN An obesity-associated FTO gene variant and increased energy intake in children N Engl J Med 2008 359 2558 2566 10.1056/NEJMoa0803839 19073975

[B5] Wardle J Carnell S Haworth CMA Farooqi SI O’Rahilly S Plomin R Obesity-associated genetic variation in FTO associated with diminished satiety J Clin Endocrinol Metab 2008 93 3640 3643 10.1210/jc.2008-0472 18583465

[B6] Vaisse C Clement K Guy-Grand B Froguel P A frameshift mutation in MC4R is associated with a dominant form of obesity Nature Genet 1998 20 113 114 10.1038/2407 9771699

[B7] Farooqi IS Keogh JM Yeo GSH Lank EJ Cheetham T O’Rahilly S Clinical spectrum of obesity and mutations in the melanocortin 4 receptor gene N Engl J Med 2003 348 1085 1095 10.1056/NEJMoa022050 12646665

[B8] Frayling TM Timpson NJ Weedon MN Zeggini E Freathy RM Lindgren CM Perry JR Elliott KS Lango H Rayner NW Shields B Harries LW Barrett JC Ellard S Groves CJ Knight B Patch AM Ness AR Ebrahim S Lawlor DA Ring SM Ben-Shlomo Y Jarvelin MR Sovio U Bennett AJ Melzer D Ferrucci L Loos RJ Barroso I Wareham NJ A common variant in the FTO gene is associated with body mass index and predisposes to childhood and adult obesity Science 2007 316 889 893 10.1126/science.1141634 17434869 PMC2646098

[B9] Loos RJ Lindgren CM Li S Wheeler E Zhao JH Prokopenko I Inouye M Freathy RM Attwood AP Beckmann JS Berndt SI Jacobs KB Chanock SJ Hayes RB Bergmann S Bennett AJ Bingham SA Bochud M Brown M Cauchi S Connell JM Cooper C Smith GD Day I Dina C De S Dermitzakis ET Doney AS Elliott KS Elliott P Prostate, Lung, Colorectal, and Ovarian (PLCO) Cancer Screening Trial Common variants near MC4R are associated with fat mass, weight and risk of obesity Nat Genet 2008 40 768 775 10.1038/ng.140 18454148 PMC2669167

[B10] Kring SI Holst C Zimmermann E Jess T Berentzen T Toubro S Hansen T Astrup A Pedersen O Sørensen TI FTO gene associated fatness in relation to body fat distribution and metabolic traits throughout a broad range of fatness PLoS One 2008 3 e2958 10.1371/journal.pone.0002958 18698412 PMC2493033

[B11] Ramos RB Casanova GK Maturana MA Spritzer PM Variations in the fat mass and obesity-associated (FTO ) gene are related to glucose levels and higher lipid accumulation product in postmenopausal women from southern Brazil Fertil Steril 2011 96 974 979 10.1016/j.fertnstert.2011.07.1148 21868005

[B12] Liu G Zhu H Lagou V Gutin B Barbeau P Treiber FA Dong Y Snieder H Common variants near melanocortin 4 receptor are associated with general and visceral adiposity in European- and African-American youth J Pediatr 2010 156 598 605 10.1016/j.jpeds.2009.10.037 20070976 PMC4018229

[B13] Li H Kilpeläinen TO Liu C Zhu J Liu Y Hu C Yang Z Zhang W Bao W Cha S Wu Y Yang T Sekine A Choi BY Yajnik CS Zhou D Takeuchi F Yamamoto K Chan JC Mani KR Been LF Imamura M Nakashima E Lee N Fujisawa T Karasawa S Wen W Joglekar CV Lu W Chang Y Association of genetic variation in FTO with risk of obesity and type 2 diabetes with data from 96,551 East and South Asians Diabetologia 2012 55 981 995 10.1007/s00125-011-2370-7 22109280 PMC3296006

[B14] Qi L Kraft P Hunter DJ Hu FB The common obesity variant near MC4R gene is associated with higher intakes of total energy and dietary fat, weight change and diabetes risk in women Hum Mol Genet 2008 17 3502 3508 10.1093/hmg/ddn242 18697794 PMC2572696

[B15] Tschritter O Haupt A Preissl H Ketterer C Hennige AM Sartorius T Machicao F Fritsche A Häring HU An obesity risk SNP (rs17782313) near the MC4R gene is associated with cerebrocortical insulin resistance in humans J Obes 2011 2011 283153 21773004 10.1155/2011/283153 PMC3136179

[B16] Pausova Z Syme C Abrahamowicz M Xiao Y Leonard GT Perron M Richer L Veillette S Smith GD Seda O Tremblay J Hamet P Gaudet D Paus T A common variant of the *FTO* gene is associated with not only increased adiposity but also elevated blood pressure in French Canadians Circ Cardiovasc Genet 2009 2 260 269 10.1161/CIRCGENETICS.109.857359 20031594

[B17] Gerken T Girard CA Tung YC Webby CJ Saudek V Hewitson KS Yeo GS McDonough MA Cunliffe S McNeill LA Galvanovskis J Rorsman P Robins P Prieur X Coll AP Ma M Jovanovic Z Farooqi IS Sedgwick B Barroso I Lindahl T Ponting CP Ashcroft FM O'Rahilly S Schofield CJ The obesity-associated FTO gene encodes a 2-oxoglutarate-dependent nucleic acid demethylase Science 2007 318 1469 1472 10.1126/science.1151710 17991826 PMC2668859

[B18] Schmid PM Heid I Buechler C Steege A Resch M Birner C Endemann DH Riegger GA Luchner A Expression of fourteen novel obesity-related genes in zucker diabetic fatty rats Cardiovasc Diabetol 2012 11 48 59 10.1186/1475-2840-11-48 22553958 PMC3398851

[B19] Guyenet PG The sympathetic control of blood pressure Nat Ver Neurosci 2006 7 335 346 10.1038/nrn1902 16760914

[B20] Cone RD The central melanocortin system and energy homeostasis Trends Endocrinol Metab 1999 10 211 216 10.1016/S1043-2760(99)00153-8 10407394

[B21] Greenfield JR Miller JW Keogh JM Henning E Satterwhite JH Cameron GS Astruc B Mayer JP Brage S See TC Lomas DJ O'Rahilly S Farooqi IS Modulation of blood pressure by central melanocortinergic pathways N Engl J Med 2009 360 44 52 10.1056/NEJMoa0803085 19092146

[B22] Timpson NJ Harbord R Davey Smith G Zacho J Tybjaerg-Hansen A Nordestgaard BG Does greater adiposity increase blood pressure and hypertension risk? Mendelian randomization using the *FTO* /*MC4R* genotype Hypertension 2009 54 84 90 10.1161/HYPERTENSIONAHA.109.130005 19470880

[B23] Ortega-Azorín C Sorlí JV Asensio EM Coltell O Martínez-González MÁ Salas-Salvadó J Covas MI Arós F Lapetra J Serra-Majem L Gómez-Gracia E Fiol M Sáez-Tormo G Pintó X Muñoz MA Ros E Ordovás JM Estruch R Corella D Associations of the FTO rs9939609 and the MC4R rs17782313 polymorphisms with type 2 diabetes are modulated by diet, being higher when adherence to the Mediterranean diet pattern is low Cardiovasc Diabetol 2012 11 137 149 10.1186/1475-2840-11-137 23130628 PMC3495759

[B24] Thomsen M Dahl M Tybjærg-Hansen A Nordestgaard BG β2-adrenergic receptor Thr164Ile polymorphism, obesity, and diabetes: comparison with FTO, MC4R, and TMEM18 polymorphisms in more than 64,000 individuals J Clin Endocrinol Metab 2012 97 1074 1079 10.1210/jc.2011-3282 22466342

[B25] Marcadenti A Fuchs SC Moreira LB Wiehe M Gus M Fuchs FD Accuracy of anthropometric indexes of obesity to predict diabetes mellitus type 2 among men and women with hypertension Am J Hypertens 2011 24 175 180 10.1038/ajh.2010.212 20885370

[B26] Pickering TG Hall JE Appel LJ Falkner BE Graves J Hill MN Jones DW Kurtz T Sheps SG Roccella EJ Subcommittee of Professional and Public Education of the American Heart Association Council on High Blood Pressure Research Recommendations for blood pressure measurement in humans and experimental animals. Part 1: Blood pressure measurement in humans – a statement for professionals from the Subcommittee of Professional and Public Education of the American Heart Association Council on High Blood Pressure Hypertension 2005 45 142 161 15611362 10.1161/01.HYP.0000150859.47929.8e

[B27] Chobanian AV Bakris GL Black HR Cushman WC Green LA Izzo JL Jr Jones DW Materson BJ Oparil S Wright JT Jr Roccella EJ Joint National Committee on Prevention, Detection, Evaluation, and Treatment of High Blood Pressure. National Heart, Lung, and Blood Institute; National High Blood Pressure Education Program Coordinating Committee Seventh report of the Joint National Committee on Prevention, Detection, Evaluation, and Treatment of High Blood Pressure Hypertension 2003 42 1206 1252 10.1161/01.HYP.0000107251.49515.c2 14656957

[B28] American Diabetes Association Diagnosis and classification of diabetes mellitus Diabetes Care 2012 35 Suppl 1 S64 S71 22187472 10.2337/dc12-s064 PMC3632174

[B29] Craig CL Marshall AL Sjöström M Bauman AE Booth ML Ainsworth BE Pratt M Ekelund U Yngve A Sallis JF Oja P International physical activity questionnaire: 12-country reliability and validity Med Sci Sports Exerc 2003 35 1381 1395 10.1249/01.MSS.0000078924.61453.FB 12900694

[B30] Bergman RN Stefanovski D Buchanan TA Sumner AE Reynolds JC Sebring NG Xiang AH Watanabe RM A better index of body adiposity Obesity 2011 19 1083 1089 10.1038/oby.2011.38 21372804 PMC3275633

[B31] Kahn HS The "lipid accumulation product" performs better than the body mass index for recognizing cardiovascular risk: a population-based comparison BMC Cardiovasc Disord 2005 5 26 36 10.1186/1471-2261-5-26 16150143 PMC1236917

[B32] Winter Y Sankowski R Back T Genetic determinants of obesity and related vascular diseases Vitam Horm 2013 91 29 48 23374711 10.1016/B978-0-12-407766-9.00002-X

[B33] Jacobsson JA Risérus U Axelsson T Lannfelt L Schiöth HB Fredriksson R The common FTO variant rs9939609 is not associated with BMI in a longitudinal study on a cohort of Swedish men born 1920–1924 BMC Med Genet 2009 10 131 139 10.1186/1471-2350-10-131 20003232 PMC2797506

[B34] Jacobsson JA Almén MS Benedict C Hedberg LA Michaëlsson K Brooks S Kullberg J Axelsson T Johansson L Ahlström H Fredriksson R Lind L Schiöth HB Detailed analysis of variants in FTO in association with body composition in a cohort of 70-year-olds suggests a weakened effect among elderly PLoS One 2011 6 e20158 10.1371/journal.pone.0020158 21637715 PMC3103532

[B35] Preis SR Massaro JM Hoffmann U D'Agostino RB Sr Levy D Robins SJ Meigs JB Vasan RS O'Donnell CJ Fox CS Neck circumference as a novel measure of cardiometabolic risk: The Framingham Heart Study J Clin Endocrinol Metab 2010 95 3701 3710 10.1210/jc.2009-1779 20484490 PMC2913042

[B36] Mangge H Renner W Almer G Weghuber D Möller R Horejsi R Rs9939609 variant of the fat mass and obesity-associated gene and trunk obesity in adolescents J Obes 2011 2011 186368 21318054 10.1155/2011/186368 PMC3026980

[B37] Nielsen S Guo Z Johnson CM Hensrud DD Jensen MD Splanchnic lipolysis in human obesity J Clin Invest 2004 113 1582 1588 15173884 10.1172/JCI21047 PMC419492

[B38] Cheung CY Tso AW Cheung BM Xu A Ong KL Law LS Wat NM Janus ED Sham PC Lam KS Genetic variants associated with persistent central obesity and the metabolic syndrome in a 12-year longitudinal study Eur J Endocrinol 2011 164 381 388 10.1530/EJE-10-0902 21147891

[B39] Povel CM Boer JM Onland-Moret NC Dollé ME Feskens EJ Van der Schouw YT Single nucleotide polymorphisms (SNPs) involved in insulin resistance, weight regulation, lipid metabolism and inflammation in relation to metabolic syndrome: an epidemiological study Cardiovasc Diabetol 2012 11 133 142 10.1186/1475-2840-11-133 23101478 PMC3507796

[B40] Parikh M Hetherington J Sheth S Seiler J Ostrer H Gerhard G Wood C Still C Frequencies of obesity susceptibility alleles among ethnically and racially diverse bariatric patient populations Surg Obes Relat Dis 2012 9 436 411 22695173 10.1016/j.soard.2012.04.004 PMC3685296

[B41] Wu C Gong Y Yuan J Gong H Zou Y Ge J Identification of shared genetic susceptibility locus for coronary artery disease, type 2 diabetes and obesity: a meta-analysis of genome-wide studies Cardiovasc Diabetol 2012 11 68 79 10.1186/1475-2840-11-68 22697793 PMC3481354

[B42] Robiou-du-Pont S Bonnefond A Yengo L Vaillant E Lobbens S Durand E Weill J Lantieri O Balkau B Charpentier G Marre M Froguel P Meyre D Contribution of 24 obesity-associated genetic variants to insulin resistance, pancreatic beta-cell function and type 2 diabetes risk in the French population Int J Obes (Lond) 2012 37 980 985 23090577 10.1038/ijo.2012.175

[B43] Kuo JJ Da Silva AA Hall JE Hypothalamic melanocortin receptors and chronic regulation of arterial pressure and renal function Hypertension 2003 41 768 774 10.1161/01.HYP.0000048194.97428.1A 12623994

[B44] Yamada Y Tsuboi K Hattori T Murase T Ohtake M Furukawa M Ueyama J Nishiyama A Murohara T Nagata K Mechanism underlying the efficacy of combination therapy with losartan and hydrochlorothiazide in rats with salt-sensitive hypertension Hypertens Res 2011 34 809 816 10.1038/hr.2011.34 21471973

[B45] Williams MJ Almén MS Fredriksson R Schiöth HB What model organisms and interactomics can reveal about the genetics of human obesity Cell Mol Life Sci 2012 69 3819 3834 10.1007/s00018-012-1022-5 22618246 PMC11114734

